# Relationship between early lung adenocarcinoma and multiple driving genes based on artificial intelligence medical images of pulmonary nodules

**DOI:** 10.3389/fgene.2023.1142795

**Published:** 2023-02-21

**Authors:** Yajun Yin, Jiawei Lu, Jichun Tong, Youshuang Cheng, Ke Zhang

**Affiliations:** ^1^ Department of Cardiothoracic Surgery, Changzhou Second People’s Hospital, The Affiliated Hospital of Nanjing Medical University, Changzhou, Jiangsu Province, China; ^2^ Department of Cardiothoracic Surgery, Affiliated Ninth People’s Hospital, Shanghai Jiaotong University School of Medicine, Shanghai, China

**Keywords:** early lung adenocarcinoma, lung nodules, artificial intelligence, biological information discipline, medical, driver genes

## Abstract

Lung adenocarcinoma is one of the most common cancers in the world, and accurate diagnosis of lung nodules is an important factor in reducing its mortality. In the diagnosis of pulmonary nodules, artificial intelligence (AI) assisted diagnosis technology has been rapidly developed, so testing its effectiveness is conducive to promoting its important role in clinical practice. This paper introduces the background of early lung adenocarcinoma and lung nodule AI medical imaging, and then makes academic research on early lung adenocarcinoma and AI medical imaging, and finally summarizes the biological information. In the experimental part, the relationship analysis of 4 driver genes in group X and group Y showed that there were more abnormal invasive lung adenocarcinoma genes, and the maximum uptake value and uptake function of metabolic value were also higher. However, there was no significant correlation between mutations in the four driver genes and metabolic values, and the average accuracy of AI-based medical images was 3.88% higher than that of traditional images.

## 1 Introduction

With the development of the staging of early lung adenocarcinoma, the detection rate of gene abnormalities increases, which plays an important role in the early occurrence of nodular lung adenocarcinoma. In recent years, with people’s understanding of health and the continuous progress of medical imaging technology, the detection rate of pulmonary nodules is increasing every year. The previous research on the treatment, efficacy and prognosis of lung cancer is of great significance. At the same time, using a variety of biological information technology, it is found that the related genes related to the pathogenesis and prognosis of lung adenocarcinoma, which laid a foundation for the study of the molecular mechanism of lung adenocarcinoma.

Many scholars have studied the relationship between lung adenocarcinoma and multiple driving genes. Aisner Dara L believed that polygene analysis was a conventional treatment for patients with advanced lung adenocarcinoma. Lung cancer variant tissue is a multi-sectoral study aimed at finding and treating cancer predisposing factors of lung cancer patients (Aisner et al., 2018). Chuang Chen-Hua, through the combination of human lung adenocarcinoma with mouse tumor barcode and unbiased gene analysis technology, found a transcription process that could enable patients to obtain metastasis and predict patient survival (Chuang et al., 2017). Chen Jianbin obtained that the genome of East Asian LUAD (lung adenocarcinoma) was more stable than that of European LUAD through research. Its characteristic was that the variation was small and the number of copies changes little (Chen et al., 2020). Gillette Michael A believed that proteome data set provided a unique shared resource for researchers and clinicians to find better understanding and treatment of lung adenocarcinoma (Gillette et al., 2020). Jamal-Hanjani Mariam believed that the tumor internal heterogeneity caused by chromosome instability was related to the increased risk of recurrence and death, which indicated that chromosome instability might be a prognostic factor (Jamal-Hanjani et al., 2017). Faruki Hawazin analyzed the molecular expression of lung AD (Alveolar Duct) and SCC (Squamous Cell Carcinoma), showing important and repeatable differences. Evaluation of tumor expression subtypes is a possible biomarker of immunotherapy (Faruki et al., 2017). Lindeman Neal I discussed the molecular analysis of lung cancer as a standard for treatment decisions of targeted inhibitors. New research led to the evaluation of alternative analysis techniques, target genes, patient populations and tumor types (Lindeman et al., 2018). The above research has achieved good results. However, with the continuous updating of technology, there are still some problems.

AI medical images are widely used in the treatment process. Pesapane Filippo used the radio diagnostic model to summarize AI science in the forensic field (Pesapane et al., 2018). Kaissis Georgios introduced a privacy based medical image analysis technology, which was an open-source software architecture that could perform joint learning and cryptographic reasoning under different private and secure aggregations (Kaissis et al., 2021). Alexander Alan believed that AI greatly changed the medical imaging market, thereby changing the working mode of imaging physicians, and helped imaging physicians speed up scanning to make more accurate diagnosis so as to reduce the workload (Alexander et al., 2020). Lee Cecilia S believed that although AI had made great progress, there were still some problems in its use in medical imaging due to the work pressure and limitations of experts’ labels (Lee et al., 2019). Allen Jr Bibb analyzed that in the field of medical imaging, the development of machine learning technology had been rapidly developed in academic and industrial laboratories. The main AI tools in diagnosis imaging include disease detection and classification, image optimization, radiation reduction and workflow improvement (Allen et al., 2019). Arabi Hossein briefly introduced the application of AI technology in molecular imaging, radiotherapy and other fields in recent years, especially in depth learning. The application of AI was discussed from five aspects: PET (polyethylene terephthalate) instrument design, PET image quantization and segmentation, image denoising, radiation dosimetry, computer aided diagnosis and prediction (Arabi and Zaidi, 2020). Nakata Norio analyzed the latest computer vision research report and selected potential clinical applications from the perspective of radiologists, such as generating confrontation networks, knowledge extraction and general image data to guide learning (Nakata, 2019). The above research shows that the application of AI medical imaging has a positive effect, but there are still some problems.

In this paper, the relationship between AI technology and various driving genes in lung tumors on lung nodules was discussed. First, the role of AI technology in early lung adenocarcinoma of lung nodules was analyzed and simulated. This article also introduced the application of bioinformatics technology to study the factors affecting the occurrence, development, and prognosis of lung adenocarcinoma, and preliminarily discussed its role.

## 2 The study design of the relationship between early lung adenocarcinoma and multiple driver genes based on medical images of lung nodules

Due to the application of high-resolution computed tomography and the early detection of small pulmonary nodules, lung cancer has become the most common pathological type. Since EGFR gene mutation, targeted therapy has greatly improved the survival rate of lung cancer, especially breast cancer, and accurate molecular phenotype is a prerequisite for tumor treatment. CT examination is the main examination method of lung adenocarcinoma. The relationship between CT images and the expression level of control genes has certain reference value for the diagnosis and treatment of lung adenocarcinoma.

Study design: the factors related to artificial intelligence medical imaging are introduced to provide a theoretical basis for the following experiments; Then, we analyzed the application of artificial intelligence medical imaging in the early stage of lung adenocarcinoma of lung nodules, further discussed how to use medical imaging to assist the treatment of lung adenocarcinoma, and finally, combined with the materials described above, we made an empirical study on the relationship between early stage of lung adenocarcinoma of lung nodules and multiple driving genes.

## 3 Overview of experimental materials and methods for clinical application of early lung adenocarcinoma

(1) General materials

A total of 50 patients with lung adenocarcinoma were selected as the study object, from Changzhou Second People’s Hospital. Among the 50 patients with lung nodules, 31 were male and 19 were female, aged 47–73 years with an average age of 60 years (60 ± 7.51).

A total of 50 patients with lung adenocarcinoma were selected as the study subjects, including 31 men of the 50 patients with pulmonary nodules, 19 women, aged 47–73 years and with a mean age of 60 years (60 ± 7.51), as shown in Table 1.

(2) Treatment
1) The preoperative computed tomography (CT) scanning results of the patient were collected. The images were retrieved from the image library, and the CT scanning results were analyzed by several senior radiologists.
2) The original HE (hematoxylin eosin) in 50 pathological specimens were sectioned for pathological diagnosis and differentiation. Two pathologists with more than the qualification of attending should perform blind examination, and the inconsistent samples were discussed again and agreed.

**TABLE 1:** Classification of patients with lung adenocarcinoma.

Patients with pulmonary nodules	Male	Female sex	Average age
Number of people	31	19	60 ± 7.51 years old
Proportion	62%	38%	100

### 3.1 The ethics committee statement

The data samples studied in this experiment are analyzed from relevant studies in this field. Animals or humans were not used as experimental subjects to promote progress in the medical field and to obtain written informed consent from the patients.

(3) Observations

It is better to have no artifact in the image, and it is better to have a small amount of artifact in the image of the tip, lung base and other parts. However, it has not any impact on the diagnostic results. The obvious artifact in the image is poor. At the same time, it also affects the internal structure and shape of the patient’s pulmonary nodules, thus making the diagnosis wrong. The results of CT examination and pathological examination of the two groups were compared and the diagnostic coincidence rate of the two groups was compared.

(4) Statistical methods

In this study, SPSS18.0 statistical software was used to process the data and average x test was used to make statistics. *p* < 0.05 indicates that there is a significant difference between the two groups.

## 4 Results of early lung adenocarcinoma with multiple driver genes

### 4.1 Factors related to artificial intelligence medical imaging

(1) The significance of AI application in medical imaging

**FIGURE 1:**
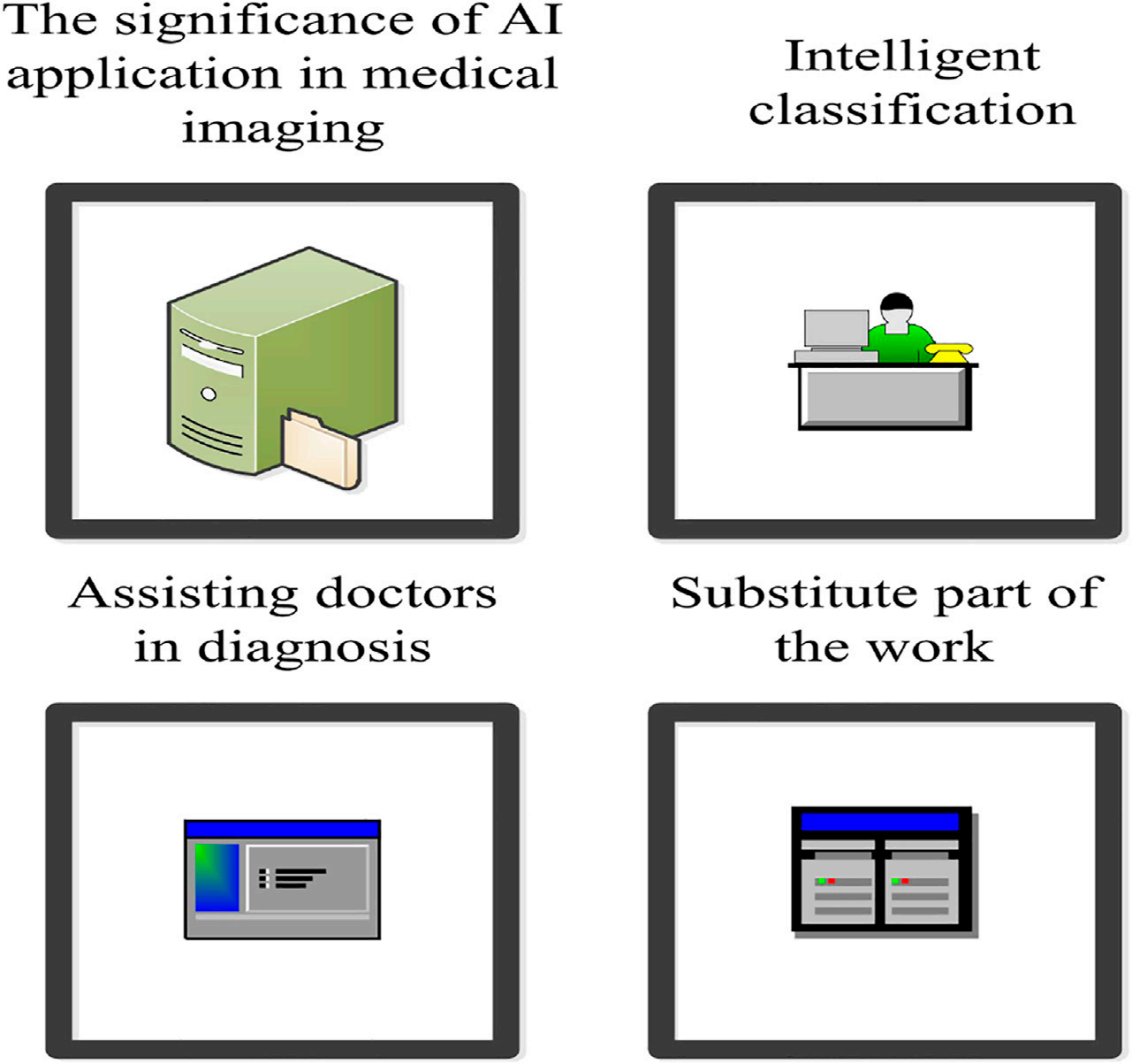
Significance of AI application to medical imaging.

This paper summarizes the significance of three-point AI in medical imaging, as shown in Figure 1.

1) Intelligent classification

AI is applicable to the type of intelligent detection. First, AI is more suitable for intelligent detection when the positive rate is low. Second, AI technology is more suitable for intelligent detection when the proportion of positive area data is low. Third, if the case image does not require too much professional knowledge, it is more suitable for AI intelligent recognition such as pulmonary nodules in physical examination. Therefore, AI technology used for intelligent classification detection can save a lot of negative case data, which can effectively reduce the work efficiency of medical personnel and reduce the waste of medical resources (Kim and Chung, 2020; Chiappelli and Penhaskashi, 2022).

2) Substitute part of the work

AI technology can replace part of the work of staff. This work can be divided into: First, if conditions are simple, it is more suitable to use AI technology to replace doctors’ work, such as robot guidance. Second, if the structure of medical knowledge required by patients is relatively simple, then AI technology can be used to replace the work of doctors, such as AI doctors. Intelligent doctors can read patients’ medical records and automatically output high-quality diagnostic results based on patients’ chief complaints, symptoms, medical history, medical images and other data. Third, when the data structure of image analysis is relatively complex, AI can be used to replace the work of some doctors, such as intelligent diagnosis of medical images.

3) Assisting doctors in diagnosis

AI technology can improve doctors’ abilities, such as auxiliary medical imaging diagnosis system. AI technology can also enhance doctors’ ears, such as intelligent medical assistants. AI technology can still enhance doctors’ brains, such as AI driven doctor assistance system (Sankaranarayanan et al., 2022).

(2) Application strategy of AI in medical image analysis

**FIGURE 2:**
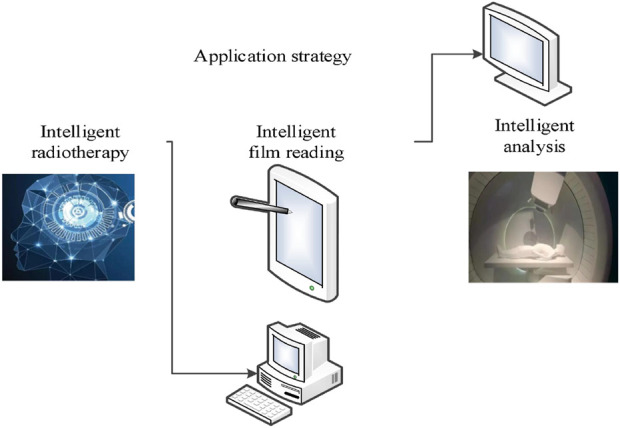
Application strategy of AI in medical imaging analysis.

This paper summarizes the application strategy of three-point AI in medical image analysis, as shown in Figure 2.

1) Intelligent film reading

AI pattern recognition can be used to predict pathological changes in medical images and help doctors identify pathological changes and improve work efficiency (Sydorova et al., 2022). Professional imaging doctors need to spend about 10 min to review the CT images of pulmonary nodules. However, AI technology is more efficient, which can quickly identify and mark small nodules. It greatly reduces the doctor’s inspection time and helps doctors make a diagnosis. In addition, the medical imaging system based on AI technology can also use the powerful learning ability of AI technology to carry out intelligent film reading and learning, so as to continuously improve the accuracy of lesion recognition. This helps doctors allocate time reasonably to better assist doctors in reading films and diagnosis.

2) Intelligent radiotherapy

At present, radiotherapy is an important method to treat cancer. Under traditional medical conditions, radiotherapy doctors are the main body of radiotherapy (Wang et al., 2022). However, due to the scarcity of radiologists, patients have to go to large hospitals. Radiotherapists need to mark the lesion manually during radiotherapy, which usually takes several hours in a conventional medical environment. With the help of AI, the lesion can be marked, which only needs to be confirmed by the radiation therapist, thus greatly improving the work efficiency.

3) Intelligent analysis

The number of pathologists in China is small. Pathologists not only spend a lot of time to make cell tissue sections, but also find small cancer cells in medical records containing billions of pixels. Therefore, even experienced case physicians makes mistakes. With the help of AI, the accuracy and efficiency of diagnosis can be greatly improved through intelligent analysis. Of course, it cannot be judged solely by AI, but also through pathological examination to avoid misdiagnosis.

### 4.2 Application of artificial Intelligence medical imaging in early lung adenocarcinoma of pulmonary nodules

(1) Application of imaging histology in lung cancer

Some scholars have studied that the appearance of surface image group can reflect the heterogeneity of tumors. Tumor type, T stage, gene expression pattern and prognosis are significantly related. Therefore, histologic imaging technology has become a hot spot in the field of lung cancer diagnosis. It has been widely used to differentiate benign and malignant pulmonary nodules and evaluate them comprehensively. This involves the analysis of histological type, cellular molecule and gene status, therapeutic effect and prognosis.

1) Screening and differential diagnosis of pulmonary nodules

The guidelines for the diagnosis and management of pulmonary nodules recommend the use of low-dose CT for screening, but the false positive rate of low-dose CT is very high. Therefore, the qualitative diagnosis of pulmonary nodules is a challenging clinical problem. Histological imaging research provides more valuable information for the screening of nodules and the differentiation between benign and malignant nodules.

2) Auxiliary pathological diagnosis

Imaging group technology can combine imaging parameters with clinical manifestations. If tumor tissue or tissue slice cannot be obtained, pathological examination by non-invasive method is helpful to determine the next treatment plan. Some studies have shown that the performance of some image groups can be used as an independent influencing factor to predict the degree of tumor invasion, so as to achieve the goal of treatment stratification for early lung cancer patients.

3) Gene expression

With the continuous development of precision medicine, the diagnosis of lung cancer patients is more and more in-depth. However, it is difficult to find the mutant gene at very low concentration. Moreover, tumor heterogeneity can be tested by biopsy. Imagomics analysis can obtain useful information about gene variation. When surgery or biopsy cannot be performed, the combination of clinical manifestations can increase diagnostic value and assist in formulating treatment plans.

4) Efficacy evaluation

It is an important basis for formulating and adjusting treatment plans to accurately estimate and evaluate the efficacy in clinical practice. Some studies have shown that the changes in the imaging group during surgery can be used as the prognosis of neoadjuvant chemotherapy to distinguish between pathological complete remission and gross residual lesions.

5) Clinical prognosis

Lung cancer is the highest mortality malignant tumor in the world today. Even if it is treated in time, a series of problems such as local recurrence, distant metastasis, radiation induced lung injury and so on appear. Therefore, it is necessary to grade high-risk patients. Imageomics has a great predictive effect on the clinical prognosis of patients.

### 4.3 Analysis of the relationship between early lung adenocarcinoma of pulmonary nodules and multiple driving genes

(1) The relationship between groups X and Y and four driving genes

The experiment showed that 50 patients with lung adenocarcinoma admitted to the hospital were collected and divided into groups. The even number is the control group (group X), and the odd number is the study group (group Y). Amplification block mutation system method was used to detect the abnormalities of A, B, C, and D (A is HER1 is also called erb B1, EGFR, B is also called erbB2, neu, C is HER3 is also called erb B3, and D is HER4 is also called er-B 4) in the samples. The relationship between driver gene mutation and pathological classification was analyzed, as shown in Table 2.

**TABLE 2:** Analysis of the relationship between groups X and Y and the four driver genes.

/	A	B	C	D	X value (%)
	(6)	(6)	(7)	(4)	
Group X	2 (33%)	2 (33%)	3 (43%)	1 (25%)	33.5
Group Y	5 (83%)	5 (83%)	5 (71%)	3 (75%)	78

The highest x value of the four driving genes in group Y was 83% and the average was 78%, which was significantly higher than that in group X. The highest X value of patients in the four driving genes was 43% and the average was 33.5%. The data were statistically significant.

(2) The relationship between abnormal driving gene and tissue type in early lung adenocarcinoma

Experiment description: 25 cases were invasive and 25 cases were non-invasive, and the analysis of A, B, C, and D driving genes was conducted by SUVmax and SUVindex analysis (SUV value is fully called standard uptake value (standard uptake value, SUV), which is a common semi-quantitative index of pet in tumor diagnosis, and refers to the radioactive activity of local tissue imaging agent and the average systemic injection activity. Invasive growth refers to the way tumor cells grow and destroy surrounding tissues, as shown in Table 3.

**TABLE 3:** Relationship between driver gene abnormalities and tissue type.

	Total	SUVmax	SUVindex	A	B	C	D
Infiltrative growth	25	7.62	4.79	0.241	0.342	0.354	0.412
Non-invasive growth	25	1.84	0.91				
Gene mutation	—						
positive	19	7.17	3.41	0.145	0.147	0.175	0.162
negative	25	6.72	4.12				

It can be seen from Table 2 that the mutation values of the four driving genes were 0.241, 0.342, 0.354, and 0.412 respectively with the change of pulmonary nodule infiltration. The highest mutation rate of D was 41.2%, and the others were C, B, A in turn. The expression of D was consistent among the four genes, and the difference was statistically significant.

(3) Image accuracy

**FIGURE 3:**
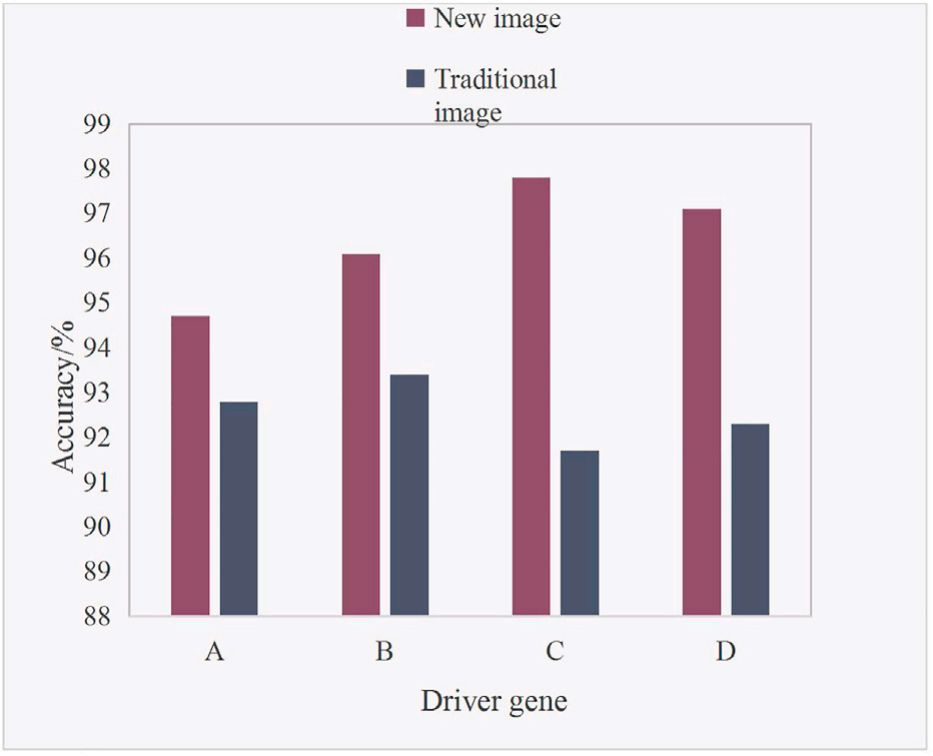
Accuracy analysis of early lung adenocarcinoma in lung nodules.

Figure 3 shows the accuracy analysis of four driving genes under AI medical imaging (new influence) and traditional imaging for early lung adenocarcinoma of lung nodules.

It can be seen from Figure 3 that the accuracy of the new image was higher than that of the traditional image in the four driving genes. The specific data analysis showed that the accuracy of the new image in driver gene A was 94.7%, and the accuracy of the traditional image in driver gene A was 92.8%. The accuracy of the new image in driver gene B was 96.1%, and the accuracy of the traditional image in driver gene B was 93.4%. The accuracy of the new image in driver gene C was 97.8%, and the accuracy of the traditional image in driver gene C was 91.7%. The accuracy of new image in driver gene D was 97.1%, and the accuracy of traditional image in driver gene D was 92.3%. It is concluded that the highest accuracy of C was 97.8% for the new image in the driver gene, while the highest accuracy of B was 93.4% for the traditional image in the driver gene. The average accuracy of the new image in the four driving genes was 96.43%, and the average accuracy of the traditional image in the four driving genes was 92.55%. The average accuracy of the new image was 3.88% higher than that of the traditional image.

(4) Effects of different imaging techniques on lung adenocarcinoma in four driving genes

**FIGURE 4:**
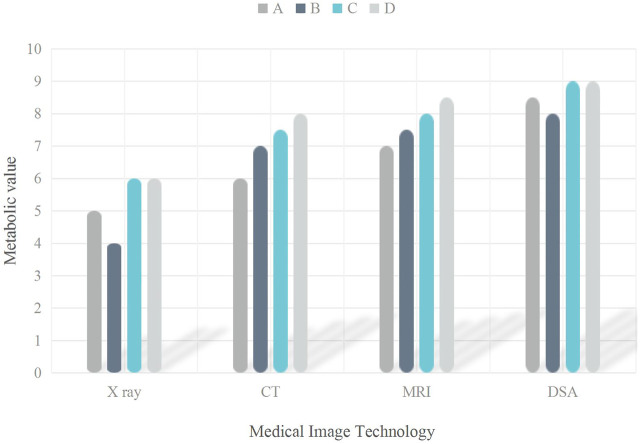
Medical image of the gene mutation in the lung adenocarcinoma in the driver gene.

Four kinds of medical imaging technologies were counted here, including X-ray, CT, MRI (Magnetic Resolution Imaging) and DSA (Digital subtraction angiography). The mutation of metabolic value in four driving genes to lung adenocarcinoma was analyzed, as shown in Figure 4.

It can be seen from Figure 4 that the metabolic values of the four medical imaging technologies in the four driving genes were different. In drive gene A, the metabolic value of X-ray was 5, that of CT was 6, that of MRI was 7, and that of DSA was 8.5. In drive gene B, the metabolic value of X-ray was 4, that of CT was 7, that of MRI was 7.5, and that of DSA was 8. In drive gene C, the metabolic value of X-ray was 6, that of CT was 7.5, that of MRI was 8, and that of DSA was 9. In driver gene D, the metabolic value of X-ray was 6, that of CT was 8, that of MRI was 8.5, and that of DSA was 9.

(5) Number of gene mutations

**FIGURE 5:**
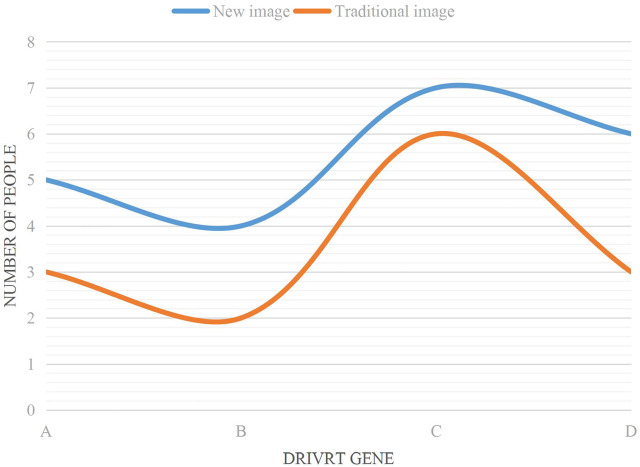
Number of gene mutations that can be detected.

Gene mutation is a tumor specific somatic genetic change. Based on the above analysis of early lung adenocarcinoma and multiple driving genes of pulmonary nodules in AI medical images, 50 patients were divided into 25 patients each. The number of people whose gene mutations could be detected was analyzed by combining new medical images with traditional medical images, as shown in Figure 5.

It can be seen from Figure 5 that the trend of the two curves of the broken line chart is from A to B, then to C, and finally to D.

According to the data analysis in the trend combination chart, 20% of the total number of 5 people were detected by the new image combined with driver gene A. Combined with driver gene B, 16% of the total number of 4 people were detected. Combined with driver gene C, 28% of the total number of 7 people were detected. Combined with driver gene D, 24% of the total number of 6 people were detected. The new image detected 88% of the total number of 22 people under four driving genes. Traditional imaging combined with driver gene A detected 12% of the total number of 3 people. Combined with driver gene B, 8% of the total number of 2 people were detected. Combined with driver gene C, 24% of the total number of 6 people were detected. Combined with driver gene D, 12% of the total number of 3 people were detected. The new image detected 56% of the total number of 14 people under four driving genes. Through calculation, the new image detected 8 more people than the traditional image.

## 5 Discussion

Science and technology have made significant progress in recent years, and the application of AI technology has provided opportunities for development in various fields, including clinical medicine. Especially in the field of medical imaging, AI technology can be used to facilitate the visualization of imaging research results. Compared with traditional manual reading, AI reading has absolute advantages, which can not only reduce the reading time but also provide a highly sensitive and accurate reference for clinical diagnosis. In this paper, 50 patients with lung adenocarcinoma were admitted and grouped, and the patients in group Y had the highest x value of 83% among the four driver genes and the average value of 78%, which was significantly higher than that in group X. Patients had the highest X value of 43% and 33.5% mean value of 34.4 driver genes.

In order to further improve the accuracy of medical image diagnosis by using AI technology, this paper studied the application of AI in medical image analysis. First, the concept and advantages of AI were introduced. Then, the importance of AI application in medical imaging was analyzed. Finally, the strategy of applying AI in medical image analysis was proposed. This paper has presented a strategy for applying AI to medical image analysis. After a period of research and practice, it is found that this strategy has universal value, because it improves the reading efficiency of medical image analysis system, which effectively improves the reading depth of medical image analysis system and helps doctors diagnose accurately.

With the deepening of scientific understanding of tumor, people gradually realize that the occurrence and development of tumor are related to multiple driver genes, so in order to better evaluate the incidence and development of lung cancer. The EGFR (epidermal growth factor receptor) collision integration method can evaluate the effect of EGFR through a single hotspot mutation such as EGFRL858R, and can predict the different expression of genes such as EGFRL858R and predict the effect of EGFR. Some scholars have conducted a single-region genome sequence analysis on many cases of lung cancer patients, and the results show that the mutation of this gene has a great relationship with the incidence of lung cancer. The prediction of NSCLC by 7-lncRNA (Long non-coding RNA) is particularly important for the early diagnosis of KRAS (Kirsten Rat Sarcoma Viral Oncogene Homolog) and EGFR.

Key driver genes and suppressor genes play important roles in the early detection and treatment of lung cancer. For early lung adenocarcinoma, a specific sequence analysis according to its clinical characteristics is combined with specific biomolecular markers to develop personalized treatment options and conduct extensive evaluation. However, as large-scale clinical data are still lacking, especially for patients with early lung adenocarcinoma, there are few data on outcome and most studies are retrospective and the follow-up time is very short. How to effectively overcome secondary drug resistance is an ongoing research topic. Furthermore, according to the Joint Committee on Cancer guidelines, many of the early-stage lung cancer studies reviewed here are rarely defined as AIS, minimally invasive adenocarcinomas, and partially appendicular growth. More attention is therefore expected to be paid to the prognosis of lung adenocarcinoma in the future.

## 6 Conclusion

In conclusion, AI based medical images are superior to traditional images in all aspects. At the same time, bioinformatics can be used to explore the pathogenesis of lung adenocarcinoma, so as to find appropriate biomarkers for early diagnosis and prognosis of lung adenocarcinoma.

## Data Availability

The original contributions presented in the study are included in the article/supplementary material, further inquiries can be directed to the corresponding authors.
